# B cells and tertiary lymphoid structures in tumors: immunity cycle, clinical impact, and therapeutic applications

**DOI:** 10.7150/thno.105423

**Published:** 2025-01-01

**Authors:** Xing Wu, Qibo Huang, Xiaoping Chen, Binhao Zhang, Junnan Liang, Bixiang Zhang

**Affiliations:** 1 Hepatic Surgery Center, and Hubei Province for the Clinical Medicine Research Center of Hepatic Surgery, Tongji Hospital, Tongji Medical College, Huazhong University of Science and Technology, Wuhan 430030, Hubei, P. R. China.; 2 Hubei Key Laboratory of Hepato-Pancreatic-Biliary Diseases, Tongji Hospital, Tongji Medical College, Huazhong University of Science and Technology, Wuhan 430030, Hubei, P. R. China.

**Keywords:** tumor immunity, B cell, tertiary lymphoid structure, atypical memory B cell, immunotherapy

## Abstract

Tumorigenesis involves a multifaceted and heterogeneous interplay characterized by perturbations in individual immune surveillance. Tumor-infiltrating lymphocytes, as orchestrators of adaptive immune responses, constitute the principal component of tumor immunity. Over the past decade, the functions of tumor-specific T cells have been extensively elucidated, whereas current understanding and research regarding intratumoral B cells remain inadequate and underexplored. The delineation of B cell subsets is contingent upon distinct surface proteins and the specific transcription factors that define these subsets have yet to be fully described. Consequently, there is a pressing need for extensive and comprehensive exploration into tumor-infiltrating B cells and their cancer biology. Notably, B cells and other cellular entities assemble within the tumor milieu to establish tertiary lymphoid structures that facilitate localized immune activation and furnish novel insights for tumor research. It is of great significance to develop therapeutic strategies based on B cells, antibodies, and tertiary lymphoid structures. In this review, we address the role of B cells and tertiary lymphoid structures in tumor microenvironment, with the highlight on their spatiotemporal effect, prognostic value and therapeutic applications in tumor immunity.

## Introduction

Tumor immunology has provided new perspectives and methodologies for cancer treatment with deepening research on the tumor microenvironment (TME). The essence of tumor immunity lies in harnessing the human immune system to recognize, respond to, and eliminate tumor cells, thereby achieving effective immune clearance of the tumor. The tumor immune microenvironment stands as a pivotal focus in tumor immunology research, encompassing intricate interactions between tumor cells and immune cells 1. Although the significance of T cells in cancer therapy and prognosis has been well established, a spectrum of strategies—from immune checkpoint blockade (ICB) therapy to adoptive T cell therapy—has revolutionized the paradigm of cancer treatment. B lymphocytes are typically less abundant and are not the spotlight in tumors, however, a multitude of recent data has indicated that tumor-specific B cells are relevant to favorable immunotherapy response and outcomes in patients.

Notably, tumor-infiltrating B cells (TIBs) are functionally diverse and heterogeneous. B cells and other immune cells are attracted to tumors and become progressively organized, evolving from the small lymphocyte aggregates into tertiary lymphoid structures (TLS). TIBs could engage in establishing immunosurveillance and potentiating antitumor immunity within the framework of tumor-associated TLS through cytokine secretion, antibody production, and antigen presentation. Nevertheless, the immunosuppressive TME, coupled with the structural disparities between assembled TLS and SLO, culminates in a compromised state of humoral immunity within TIBs and TLS. Leveraging engineered B cells, antibodies, and biocompatible TLS to reconfigure the TME may architect pioneering approaches in cancer therapy. Herein, we delve into the burgeoning roles of intratumoral B cells and TLS in immunity cycle, and illuminate their profound impact on patient prognosis and cancer treatment.

## Development and function of B cells and tertiary lymphoid structures

B-cell development is coordinated by relevant transcription factors in specific locations and environments. Hematopoietic stem cells (HSCs) upregulate transcription factors such as Early B-cell factor 1 (EBF1), Forkhead box O1 (FOXO1), Paired box 5 (PAX5), and Interferon regulatory factor 4/8 (IRF4/8), leading to lineage commitment and the generation of common lymphoid progenitors (CLPs). Through the acquisition of CD19, CD20, and BCR, progenitor B cells (pro-B cells) develop into precursor B cells (pre-B cells), and eventually immature B cells 2, which are terminally activated in the periphery through T-cell dependent and T-cell independent manners. B cells undergo further proliferation and activation within SLO and TLS. Terminally differentiated plasma cells and memory B cells execute their function through antibody production to neutralize antigens and drive antibody-dependent cellular phagocytosis (ADCP), cellular cytotoxicity (ADCC) as well as complement-dependent cytotoxicity (CDC). Recent studies have indicated that B cells and antibodies play dual roles in tumor immunity.

### Origin and differentiation of B cells in the bone marrow

B cells, including B1 and B2 cells, mediate the immune effects of antigen presentation, secretion of cytokines, and production of specific antibodies in infections, autoimmunity, and tumor diseases. CD5^+^ B1 cells, derived from fetal liver or hematopoietic stem cells, dominate the development of B cells in the late embryonic and early fetal stages, and underlie the immune responses of mucosa and epithelial tissue in the early stages of individuals. B cells are often referred to as CD5^-^ B2 cells. B2 cells originate from HSCs and serve as important components of humoral immunity during late fetal development and after birth. B cells initially differentiate and develop into pro-B cells and begin to express Igα/Igβ heterodimers. Pre-B cells come to express a µ heavy chain and form the pre-BCR complex after the Pro-B cells complete the VDJ gene segment rearrangement. Through negative selection, they generate immature B cells with mlgM and gain central tolerance through clonal deletion, receptor editing, and anergy. Finally, immature B cells that start to express mlgD, gradually egress into the bone marrow and enter the blood circulation to become transitional B cells.

### Immune response of B cells in the marginal zone, germinal center and extrafollicular region

**Figure 1:**
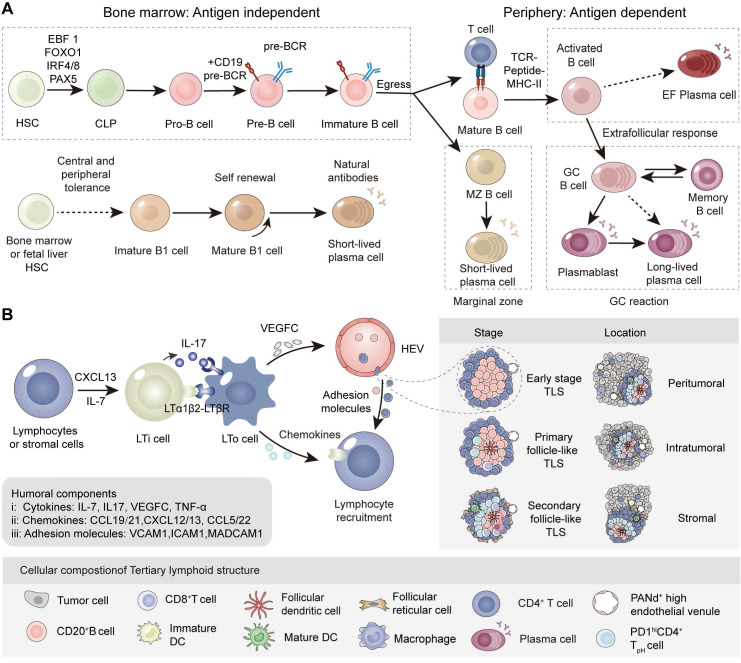
B cell development and genesis of tertiary lymphoid structures. (A) The development and maturation of the B1 lineage and B2 lineage cells. (B) Formation, maturation and location of classic TLS in tumors. In the context of long-term inflammation or high tumor immunogenicity, tissue stromal cells or lymphocytes (LTo cells) produce numerous inflammatory factors (TNFα and IL-7) and chemokines (CXCL12/13, CCL19/21 and CCL5/22). The recruited LTi cells subsequently interact with stromal cells through the LTα1β2-LTβR axis, prompting the release of VEGFC and formation of HEV. In addition, released IL-17 promotes the release various chemokines of LTo cells, which recruit lymphocytes to express LTα1β2. The adhesion molecules secreted by vascular endothelial cells, as well as chemokines, further participate in the genesis of TLS by recruiting lymphocytes through HEV. The maturity, location, and density of TLS directly affect the efficiency of immune response. Based on the degree of maturity, TLS can be divided into early, primary follicular, and secondary follicular TLS. Early TLS are composed of dense lymphocyte aggregates and lack FDCs. Primary follicular TLS begin to appear with CD21^+^CD23^-^ FDCs without GC formation, while TLS with high GC activity and CD21^+^CD23^-^ FDCs are presented as secondary follicular TLS. TLS can be divided into intratumoral, peripheral infiltrating, and peritumoral based on their distribution within the tumor. TLS provide a local and vital microenvironment for the immune response in tumors. LTi: lymphoid tissue inducing cell; LTo: lymphoid tissue organizer cell; HEV: high endothelial vein; VEGFC: vascular endothelial growth factor C.

In the peripheral lymphoid organs, transitional B cells further develop into mature B cells. It has been reported that 20% of circulating naïve B cells remain self-reactive and experience peripheral tolerance, fortunately some of which can reparticipate in immune responses through clonal redemption 3. According to their distinct maturation sites, mature B cells are sorted into marginal zone B cells (MZ BCs), follicle-germinal center cells (GC BCs), and extrafollicular B cells (EF BCs). Finally, B cells are activated, proliferate, and differentiate into plasmablasts, plasma cells, and memory B cells. The activation of B cells involves two pathways: T-cell dependent and T-cell independent pathways in the peripheral lymphoid organs (Figure 1A).

In the classical T-cell dependent pathway, B cells recognize the antigen presented by follicular dendritic cells (FDCs) through the BCR. T follicular helper (Tfh) cells stimulated by the same antigen interact with B cells at the edge of the T-cell zone and provide CD40L and cytokines for B-cell activation. Activation of the T-cell dependent pathway depends on interactions among FDCs Tfh and B cells. Ki67^+^CD23^+^ B cells undergo rapid proliferation in the dark zone of the germinal center accompanied by somatic hypermutation (SHM). Subsequently, B cells proceed to antibody class switching and affinity maturation in the bright zone, transforming from the secreting IgM type to IgA, IgE and IgG isotypes. This is essential for the different types of antigens reactions. Cytidine deaminase primarily expressed in activated B cells, is vital for the SHM and CSR processes and is involved in the formation of GC.

T-cell independent activation requires BCR to aggregate on the surface of B cells to cross-link with repeated antigen epitopes, while Toll-like receptor (TLR) stimulation provides a second activation signal. Activated B cells generate short-lived plasma cells that secrete IgM. T-cell independent pathways primarily occur in MZ BC and B1 BC (naïve-like B cells), which are important for rapid and early responses to drainage area pathogens such as blood and cavity pathogens 4.

### B Cell mobilization center in the peripheral: SLO and TLS

Tertiary lymphoid structures, also known as tertiary lymphoid organs or ectopic lymphoid structures, are organized immune cell aggregates acquired from ectopic lymphoid tissues under non-physiological conditions 5. TLS usually occur in chronic infectious tissues, autoimmune diseases and cancer. TLS have an organization similar to that of secondary lymphoid organs, including T cell zones, B cell follicles and high endothelial veins (HEV), but lack an encapsulated structure and afferent/efferent lymphatic vessels 6. There are various reports on the formation of tumor-associated TLS, stemming from studies on ectopic lymphoid tissues or organs in tumors. The initial stage of TLS formation partially depends on the secretion of IL-7 and CXCL13 by stromal cells and lymphocytes at the site of inflammation or high tumor mutation burden (TMB) 7, supporting the recruitment of lymphoid tissue inducing cells (LTi cells) such as Th17 cells, B cells, macrophages and innate lymphoid cell-3 (ILC3s) 8. Subsequently, LTi cells interact with lymphoid tissue organizer cells (LTo cells) via the LTα_1_β_2_/LTβR and IL-17/IL-17R axes, secreting a variety of effector molecules involved in peripheral node addressin-positive (PNAd^+^) HEV formation, lymphocyte recruitment (CXCL12/13, CCL19/21 and adhesion molecules) and ultimately TLS assembly 9. Although the mechanism of tumor-associated TLS formation is still controversial, the main research is analogous to the formation of SLO. It has been demonstrated that the microbiota present within the TME influences the formation of TLS 10 when solid tumors develop on barrier surfaces including lung or colorectal tissues (Figure 1B) 11. Intriguingly, recent reports have described a TLS with distinct architecture, wherein B cells are positioned at the periphery of the TLS.

Notably, the abundance, functional state, and distribution of B cells in TME are highly dependent on the location, density, and maturity of tertiary lymphoid structures 5, typically disseminated in the intratumoral and peritumoral sites. Intriguingly, immature TLS are more common than TLS with GC 12. Studies have shown that B cells prefer to form a suppressive TME in immature TLS, while mature TLS in tumors usually serve as hubs of antitumor immunity 13. These findings hold promise as a complementary approach through inducing the mature and high-density TLS in cancer treatment.

## B lymphocytes in cancer immunity cycle: Intratumoral cycle and humoral circulation pathway

Current studies have concluded the immune effects of tumor-infiltrating T lymphocytes (TILs) within the cancer immunity cycle 14. Our research group become intrigued by the source, tumor site, and distribution of tumor-infiltrating B cells. In the following section, we dissect the source of TIBs and distinguish them from TILs. Naïve B and naïve T cells within the tumor-associated TLS are generally thought to be derived from PNAd^+^ HEV 15, whereas the originally activated T lymphocytes and B lymphocytes in lymph nodes have the potential to migrate to the CD31^+^CD34^+^ tumoral vasculature. TIBs are either accumulated in the margin of tumors 16 or assembled from less organized lympho-myeloid aggregates (LMAs) to structured TLS. Next, we attempt to provide a detailed description of the spatial immune effects of the response patterns of TIBs (Table 1).

### Intratumoral cycle activation pathways based on tertiary lymphoid structures

The intratumoral immunity cycle of B cells is mainly based on TLS and lympho-myeloid aggregates. Owing to the notable differences in the therapeutic prognosis of these two pathways, we categorize the intratumoral immunity of B cells into GC pathway and EF pathway 17. Activated T and B cells in these pathways eventually participate in the killing of tumor cells and accompanied by the release of tumor antigens, which can be regarded as the initiation of intratumor immunity.

**Figure 2:**
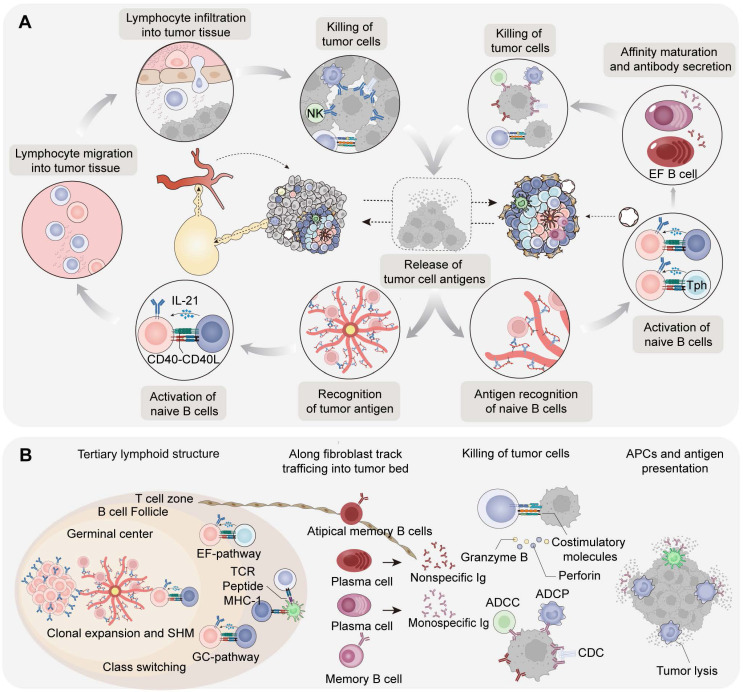
B cells in antitumor immunity: humoral circulation and local priming. (A) Schematic diagram of the B cells in cancer immunity cycle. (1) Intratumoral and circulating antibodies assist effector T cells in killing cancer cells through the immune response of neutralization, opsonization, ADCC, and CDC. (2) The release of tumor antigens or antigen-antibody complexes are taken up by APCs in tumors and are presented to lymphocytes in LMAs. (3) Next, APCs migrate to tumor-draining lymph nodes or TLS. (4) Naïve B cells in TDLNs are activated with the assistance of FDCs and CD4^+^ Tfh, and finally differentiate into affinity-matched plasma cells and memory B cells following SHM and CSR. (5) In addition, lymphocytes can migrate to tumor tissues through the circulation system under specific chemokines and adhesion molecules; antibodies secreted by plasma cells can diffuse throughout the body and bind to tumor tissue. (6) Lymphocytes infiltrate tumor tissues through HEV, tumor blood vessels and stroma, recognizing, secreting effector molecules, and killing tumor cells. (B) Intratumoral cycle activation pathway based on tertiary lymphoid structures. Compared with the GC pathway, naïve B cells are activated with the help of FDCs and CD4^+^ Tph in EF pathway and then gradually differentiate into atypical memory B cells and short-lived plasma cells. The short-lived plasma cells secrete mostly meaningless, low-affinity and self-reactive antibodies

The main function of TLS-GC is to produce memory B cells and long-lived plasma cells that secrete high affinity antibodies in tumors. In GC pathway, CXCL13^+^ Th cells in nasopharyngeal carcinoma (NPC) and breast cancer 18 are reported involving in the recruitment of GC B cells and CXCR5^+^ Tfh cells in tumor-associated TLS 19. In addition to mature dendritic cells and macrophages, TIBs can function as antigen-presenting cells (APCs) by presenting antigens to T cells, thereby promoting the activation and proliferation of CD4^+^ and CD8^+^ T cells 20. Furthermore, TLS associated follicular regulatory T cells (Tfr), regulatory B cells (Bregs), and regulatory T cells (Tregs) participate in the regulation of B and T cell activation by secreting multiple cytokines 10,21 (Figure 2B).

Recent studies have revealed that the extrafollicular (EF) pathway of B cells is independent of GC and enhanced in partial cancer 22. The precursors of antibody-secreting cells (ASCs) in the EF pathway exhibit an atypical memory B-cell phenotype (AtM B cells). Further analysis revealed that the EF pathway is dominant in hepatocellular carcinoma (HCC), pancreatic adenocarcinoma (PDAC), and cervical squamous cell carcinoma. Both AtM B cells in the EF pathway and classical memory B cells in the GC pathway originate from naïve B cells, whereas T helper cells in the GC and EF pathways are distinct. T peripheral helper (Tph) cells in EF pathway are involved in inducing the formation of AtM B cells and show an exhaustive phenotype 23. Naïve B cells mediate the activation of EF pathway B cells under the auxiliary stimulation of PD-1^hi^CXCL13^+^CXCR5^-^ Tph from the T-cell area and tumor antigen complexes presented by FDCs. ASCs ultimately differentiate into AtM B cells and short-lived plasma cells which secrete low-affinity antibodies and begin to migrate away from TLS. In spatial TME architecture of renal cell carcinoma (RCC) patients, plasma cells generated in tumor-associated TLS were observed to further disseminate into the tumor bed along CXCL12^+^ fibroblastic tracks 24. It can be speculated that the EF pathway and functional exhaustion of AtM B cells are upregulated in tumors, seen as an adaptive response or metabolic reprogramming.

### Humoral circulation activation pathways based on secondary lymphoid organ

The humoral circulation activation pathway, especially in tumors that lack mature TLS, serves as another pathway of immunity cycle in B cells. Evidence suggests that B cells engage in a systemic response to tumor antigens, as indicated by tumor-specific B cells, plasma cells and antibodies detected in the peripheral blood 25. Based on previously reported tumor antigens found in the serum, tumor-specific B cells in peripheral lymphatic organs may be selectively activated and produce antibodies when tumor antigens are released into the blood or presented by APCs through lymphatic vessels 26. These steps allow lymphocytes to proliferate and differentiate into effector T cells and memory B cells, which migrate into the tumor bed and lead to the destruction of tumor cells 27. It has been hypothesized that activated plasma cells may even migrate to the bone marrow for long-term survival and antibody production under chronic stimuli. The antibody continuously secreted by plasma cells in the SLO will eventually spread to the tumor bed through lymphatic and blood circulation.

In particular, B cells in peripheral lymphoid tissue and circulation may be attracted directly to the tumor bed instead of exclusively through HEV of TLS, despite the fact that they are less abundant in tumors without TLS 28,29. In TLS-deficient tumors, it has been postulated that B cells can be recruited through conventional CD31^+^CD34^+^ blood vessels 21. Tumor-infiltrating immune cells and fibroblasts in tumor tissues could secrete chemokines such as CXCL13 to produce chemotactic effects on B cell subsets. In addition, CXCL9 and CXCL10 secreted by the intestinal epithelium can participate in the recruitment of IgA^+^ plasma cells in mucosal tumor such as colorectal cancer 30. Interestingly, owing to the lack of effective vascular systems in solid tumors, circulating antibodies and immune cells may only penetrate the superficial layer of the tumor, which is consistent with the vascular distribution of the tumor 31. Therefore, TLS-based intratumoral immunity may be a strategy for the penetration of immune cells to antagonize the nutrient-poor and the oxygen-deficient tumor cores (Figure 2A).

## Critical subsets of tumor-Infiltrating B cells in TME

Subsets of TIBs and antibodies have attracted increasing attention in cancer research and clinical practice. Several studies have focused on profiling the immune landscape of B cells and plasma cells across distinct cancers through sequencing technology 22,32. The imbalanced status is revealed in intratumoral regulatory B cells and functional B cells including the components of antitumor plasma cells and memory B cells, induced by immunosuppressive TME.

### Regulatory B cells in TME

**Figure 3:**
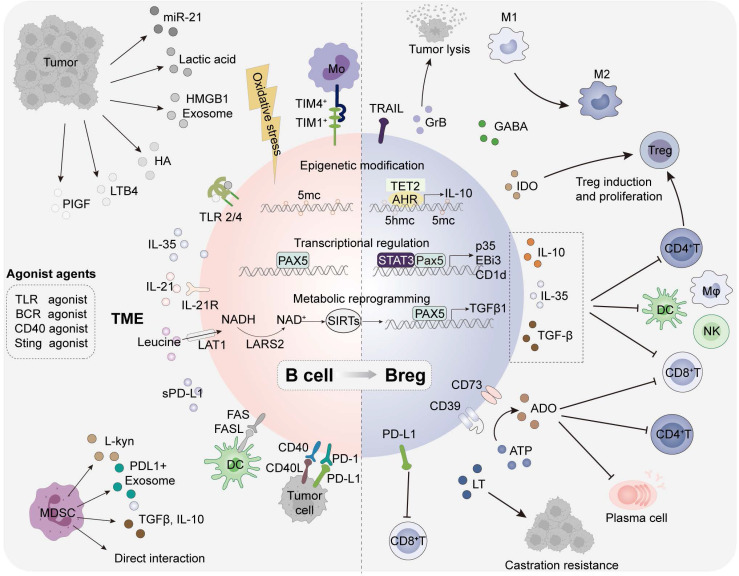
Functional B cells and regulatory B cells in the tumor microenvironment. Components in TME could participate in tumor-induced regulatory B cells through epigenetic modifications, transcriptome regulation, and metabolic reprogramming. HA, LTB4, lactate, and exosomes can promote transfer of B cells into regulatory phenotypes by acting as BCR agonists, TLR agonists, CD40 agonists or Sting agonists. Bregs can exert regulatory functions by upregulating impressive surface markers and the secretion of multiple inhibitory cytokines. ROS: reactive oxygen species; PlGF: placenta growth factor; L-kyn: L-kynurenine; LTB4: leukotrienes B4; HA: hyaluronic acid; HMGB1: high mobility group box 1; AHR: aryl hydrocarbon receptor; PPARα: peroxisome proliferator-activated receptor α; TIM: T-cell immunoglobulin and mucin-domain; MDSC: myeloid-derived suppressive cell; LT: lymphotoxin; TRAIL: TNF-related apoptosis-inducing ligand; GABA: γ-Aminobutyric Acid; ADO: adenosine; GrB granzyme B; IDO: indoleamine-2,3-dioxygenase; 5-LO: 5-lipoxygenase.

The role of regulatory B cells has been widely reported in infections, autoimmune diseases, transplantation, allergic diseases and tumors. Bregs, originally identified in autoimmune enteritis, are a subset with immunosuppressive function that play an important role in individual immune homeostasis 33. However, the specific transcription factors associated with Bregs have not been clearly characterized, posing challenges for research in this field. It still remains controversial whether Breg constitute an independent subset or a regulatory B-cell state induced under distinct conditions. Supporting this view, it is currently found that immature B cells 34,35, plasma cells 30, plasmablasts 36 and even B1 cells 37,38 all have the potential to differentiate into Bregs and produce IL-10. In this section, we introduce the cytokines and metabolites involved in Breg induction, intracellular biological pathways and Breg effector functions in tumors (Figure 3).

Indeed, Bregs with multiple surface markers have been identified in human and mouse tumors, peripheral blood, and draining lymph nodes (Table 2). TME derived soluble molecules (IL-1β 39, IL-21 40,41, IL-35 42, ROS 43, PlGF 44, miR-21 45, and sPD-L1 46), metabolites (Leucine 47, L-kyu 48, LTB4 49, hyaluronan 50, and lactic acid), exosomes 51 (PD-L1 52 and HMBG1 53) and cell-interactions have been reported as involving in the induction of Bregs. Given that most Bregs are induced at different B cell stages, it is particularly critical to explore and evaluate the environmental factors that drive Breg production. These molecules can promote Breg differentiation by binding to specific receptors (IL-21R 40, AHR 48, PPARα 49, TLR2/4 53 and BCR 54) of B cells. Intriguingly, many receptor agonists (BCR agonists 55, TLR agonists 50,53 CD40 agonists 56 and STING agonists 57) have been unveiled in B cell activation but may also trigger Breg differentiation in some cases, possibly related to the stimulation duration and concentration of these agents. In addition, multiple contact-dependent mechanisms have been uncovered in Breg generation, including CD40/CD40L and PD-1/PD-L1 by tumor cells, TIM-1/TIM-4 by CD11b^+^ myeloid cells, CD95/CD95L by semimature DCs, PD1/PDL1 by MDSCs.

The total external stimulation triggers B cell intracellular epigenetic modification, transcriptional regulation and metabolic reprogramming, mediating Breg transformation. Triggered by oxidative stress in the HCC microenvironment, a predominant epigenetic regulatory enzyme ten-eleven translocation-2 (TET2) could promote the generation of Bregs by catalyzing the formation of 5-hydroxymethylcytosine (5-hmc) and enhance IL-10 transcription in B cells 43. The dysregulated transcriptional program impeding plasma cells differentiation in PDAC involves an IL-35-induced STAT3-PAX5 signaling pathway that upregulates BCL6, promoting the transformation of naive B cells into Bregs and contributing to immune evasion 42. In addition, leucine-rich diets associated leucine-tRNA-synthetase-2-expressing (LARS2^+^) Bregs in colorectal cancer (CRC) can lead to the TGF-β1 production and immunosuppression through mitochondrial NAD^+^ regeneration and oxidative metabolism 47.

Tumor induced Bregs resemble Tregs can participate in the formation of an immunosuppressive TME through the secretion of various effector molecules and the upregulation of surface markers 58. The suppressive patterns are mainly divided into three types: conventional effector molecules (IL-10, IL-35, TGFβ, LT, PD-L1, FasL and TRAIL), plasma cell-related effector molecules (IgA and IgG), neurotransmitters and metabolites (GABA, ADO, GrB and IDO) 41. They can inhibit the function of anti-tumor immune T cells and natural killer (NK) cells, promote the generation of Tregs, and suppress the effects of APCs through various mechanisms to potentiate tumor growth, proliferation, and immune evasion 36. Bregs have been implicated in poor prognosis and drug resistance across various tumor types. The impact of TIBs can be either anti-tumorigenic or pro-tumorigenic, contingent on the specific malignancy and the equilibrium between functional B cells and regulatory B cells. A more detailed characterization of Breg subsets may offer significant advancements for cancer research.

### Plasma cells and antibodies in TME

Tumor-infiltrating plasma cells are a group of highly heterogeneous cells and humoral immunity status dominates in distinct anti-tumor response. In poorly immunogenic tumors, plasma cells and antibodies form immune complexes that serve as an amplification loop and downregulate the antigen threshold required for T cell activation, enhancing T cell responses against tumor cells 59.

Although tumor-specific plasma cells selectively expand under the stimulation by tumor antigens, the effective clonal expansion is far less than the number of potential tumor neoantigens 60. The contradictory situation of mutation-generated tumor antigens and effectively responsive B cells reflects impaired humoral immunity in tumors, which may be partially attributed to the suppressive microenvironment or the difference between tumor-induced TLS and well-structured lymph nodes. Tumor-associated antigens (TAAs) are usually described as highly expressed self-antigens including cancer germline antigens, while tumor-specific antigens (TSAs) are classified as genomic mutations and posttranscriptional aberrations of normal proteins. Mechanistically, this may also elucidate the coexistence that self-reactive plasma cells and auto-antibodies have been found in tumor tissues and peripheral blood 61, rendering either anti-tumor or pro-tumoral humoral responses. Coincidentally, two types of related growth factor receptor auto-antibodies isolated from B cells show opposite effects, with agonist antibodies promoting the growth of breast cancer cells and antagonizing the opposite of that in breast cancer patients 62. In addition to antibodies targeting tumor neoantigens, there are also specific antibodies against microbial proteins in the context of viral infection-related tumors 63.

Intratumoral TLS are sites for the generation of mature B cell immunity, where plasma cells can migrate into the tumor bed along the fibroblastic tracks and secrete IgG or IgA antibodies 24. IgG^+^ plasma cells, which are typically associated with improved clinical outcomes are preferentially present in inflamed tumors, while IgA^+^ plasma cells are predominant in mucosal epithelial tumors 64. To be specific, it is found that glucose deprivation may suppress IgG^+^ plasma cell generation and antitumor immunity through the SATB1/STAT6 axis in colorectal cancer 65. In addition, CXCL10-induced macrophages and IgG^+^ plasma cells contribute to the immunosuppressive microenvironment in HCC 66. Furthermore, IgG^+^ plasma cells targeting heat shock protein 4 (HSPA4) in tumor-draining lymph nodes (TDLNs) participate in the formation of the premetastatic niche in breast cancer 67. In contrast, IgA^+^ plasma cells expressing IL-10 or programmed death ligand 1 (PD-L1) dismantle the antitumor immunity of T cells in HCC and prostate cancer, whereas they are involved in the protective humoral response in ovarian cancer 58,60,68.

### Memory B cells in TME

Human memory B cells, defined as classical class-switched CD21^+^CD27^+^ B cells and CD21^-^CD27^-^ atypical memory B cells, are the reserve force of the immune responses and are more abundant than naïve B cells in the peripheral blood, especially in elderly individuals 69. On the basis of the surface markers IgD and CD27, IgD^-^CD27^-^ refers to double-negative B cells (DNBs). This section primarily introduces AtM B cells in tumors owing to the space limitations.

AtM B cells, characterized as CD11c^+^CD21^-^CD27^-^IgD^-^Tbet^+^ in human, are upregulated during aging, chronic infections, and autoimmune diseases and accumulate more significantly in female. The transcription factors T-bet and Zeb2 may be vital components of AtM B cells to achieve antibody class switching and antigen presentation 70. They express immunosuppressive FCRL4, cytokine IL-10 and receptors, typically participating in the inhibition of anti-tumor response. As mentioned earlier, regardless of whether glutamine metabolic derivatives or Tph interacts, the transcription factor Zeb2 may be necessary for the formation of AtM B cells in tumors. Zeb2 can mediate the transformation of GC B cells to EF AtM B cells by downregulating Mef2b expression, thereby mediating the exhaustion of phenotypic B cells in tumors. An in-depth exploration of the molecular mechanism through which Zeb2 promotes the occurrence of AtM B cells, may present alternative ideas and outlooks on cancer research.

A single-cell B cell profiling reveals stress-responsive memory B cells and tumor-associated atypical B cells, two tumor-rich subsets with prognostic potential that are shared in a pan-cancerous manner. In particular, tumor-associated AtM B cells are characterized by high levels of clonal expansion and proliferation, as well as close interactions with activated CD4^+^ T cells in tumors, which can predict the immunotherapy response 32. Another tumor-infiltrating B cell sequencing study demonstrated that atypical memory B cells developed by EF pathways, are independent of TLS-GC B cells and enriched in tumors, which are associated with shorter overall survival (OS) in patients 22. An immunosuppressive AtM B cell subset was identified in a mouse model of bladder cancer, and depletion of this subset delays cancer progression, indicating that AtM B cells promote bladder cancer progression 71. AtM B cells exhibit an exhausted phenotype and are increased in non-small cell lung cancer (NSCLC) and mesothelioma patients with non-response to ICB therapy, suggesting that AtM B cells may impede the ICB treatment response 72. In addition, AtM B cells are enriched in the peripheral blood of patients with breast cancer compared with healthy controls, and their role needs to be further elucidated 73.

## The crosstalk between TIBs and other tumor infiltrating immune cells

### The interaction between B cells and T cells in TME

TIBs engage in intricate interactions with resident immune cells and cancer cells, which affects tumorigenesis and progression. The presence of TIBs close to CD8^+^ T cells or the co-localization of B cells and T cells in the intraepithelial infiltration of breast cancer, malignant melanoma and ovarian cancer are positive prognostic markers 74. The close contact between TILs and TIBs indicates a functional interaction among them, which is related to enhanced local immune activation and helps to improve the prognosis of patients 75. However, the coexistence of Bregs and Tregs in TIL aggregates is correlated with shorter metastasis-free survival in cancer patients 76. Cho *et al*. indicated that impaired Tfh-B-Trm (tissue-resident memory T cell) cooperation in the formation of TLS, accompanied by dysregulated Trm homeostasis and loss of Tfh-B crosstalk, underlies the unfavorable anti-PD-1 response in EGFR-mutated lung cancer 77. In a breast cancer mouse model treated with low-dose cyclophosphamide combined with CSF1R inhibitors, CD4^+^ T cells and antigen-presenting B cells were enriched and colocalized in the TLS, inducing the durable tumor regression after combination treatment 78.

Specifically, three types of CD4^+^ TIL responses to TIBs have been identified: activated, antigen-related and unresponsive TIBs. In the activated antigen-related CD4^+^ TIL population, activated TIBs are associated with the effector T cell response. Alternatively, exhausted TIBs are associated with the regulatory T cell phenotype 79, suggesting that targeting TIBs can serve as the potential therapeutic strategy for immunotherapy in NSCLC. Tumor neoantigens recognized by TILs promote the crosstalk between tumor-specific CD4^+^ T cells and tumor-specific B cells, thus enhancing the anti-tumor immunity of CD8^+^ T cells in lung adenocarcinoma (LUAD) 80. Recent preclinical studies have shown that the generation of IL35^+^ B cells induced by STING agonists may attenuate the proliferation of NK cells and weaken the NK-driven anti-tumor response. It is suggested that the combination of inflammation-inducing STING agonists and IL-35 blockers may optimize this outcome 57.

### The interaction between B cells and myeloid cells in TME

The crosstalk between tumor-infiltrating B cells and myeloid cells (macrophages, DCs, MDSCs, and neutrophils) has also been reported. Apart from direct cell-cell contact, soluble metabolites derived from B cells can perform immunomodulatory functions, which may be drug targets of the immune responses. B cells and plasma cells can secrete metabolites GABA, promoting the differentiation of monocytes into anti-inflammatory macrophages, which can secret IL-10 and inhibit the killing function of CD8^+^ T cells 81. Besides, during cancer treatment, the macrophage layer of the subcapsular sinus in TDLNs is undermined, allowing tumor-derived extracellular vesicles to interact with B cells of lymph node cortex and mediate cancer-enhancing immunity 82. IL17^+^ Th cells selectively recruit CXCR3^+^ B cells which can induce tumor-promoting macrophage polarization and facilitate hepatocellular carcinoma development 83.

Moreover, immune complexes deposited in precancerous tissue, promote squamous cell carcinogenesis by activating bone myeloid cells dependent on Fcγ receptors. More than 50% of B cells display an FcγRII^low/-^ activation phenotype in HCC, and this phenotype is positively correlated with cancer progression. Further studies have revealed that semimature dendritic cells in tumors participate in the formation of FcγRII^low/-^ B cell dependent on the CD95L pathway. FcγRII^low/-^ B cells can secrete IL-10 to inhibit the anti-tumor response of cytotoxic T cells 84. MDSCs, accumulated around the GC in the spleen of tumor-bearing mice, could promote B-cell production of IgA in a TNFR2-dependent manner 85. Tumor-induced Bregs (tBregs) can initiate the regulatory subset functions of MDSCs and suppress antitumor immunity, which is partially dependent on TGFβR1/TGFβR2 signaling 86. There is evidence that tumor-associated neutrophils drive B cell recruitment through the release of TNF-α and regulate plasma cell differentiation through neutrophil membranal B-cell activating factor (BAFF) 87.

## The prognostic and predictive value of B cells and TLS in human cancer

The genetic traits of cancers, comprising tumor mutational burden, immune checkpoint expression and immune cell infiltration, are critical indicators of response to immunotherapy in patients with tumors 88. The manifestation of TLS, along with intratumoral B cells, has been reported supporting the favorable outcomes and ICB response in both immunogenic tumors and TMB-low tumors 10. Investigating the composition of tumor-associated TLS and TIBs can shed light on their contribution to the prediction and prognosis of cancer patients, supporting therapeutic approaches focused on B cells and TLS.

### Methods for TIBs and TLS detection and evaluation in tumors

To fully characterize TIBs and TLS in tumors, it is critical to employ proper techniques to identify and analyze their signatures in the TME 27. B cell detection and quantification can be achieved through methods such as flow cytometry, transcriptomic sequencing profiles, and immuno-imaging techniques. Conventional methods for detecting TLS rely on surgically obtained tumor tissue samples and include hematoxylin and eosin (H&E) staining, immunofluorescence (IF), multiplex immunohistochemistry (mIHC), RNA sequencing (RNA-seq), and spatial transcriptomics (ST) 89. In addition, histological and genomic analyses can be applied to quantitatively evaluate TLS. Compared with the histological analysis, integrating multi-omics approaches may be further put into use to effectively and complementarily assess TLS in the future. The 12-chemokine signature (12-CS) has been identified to characterize the gene expression profiles of TLS 90, which connects a specific set of chemokine receptor/ligand genes with the formation and maintenance of TLS in tumors. The application of nanomaterials and artificial intelligence has driven the development of non-invasive imaging based on nanoprobes, such as CT and MRI 91. These non-invasive imaging techniques provide new insights into the predictive and prognostic value of TLS 92.

### The prognostic value of TIBs and TLS in patient survival

Previous reports have pointed out that the heterogeneity of tumor-infiltrating B cell subsets determines their dual roles of pro-tumor and anti-tumor. In preclinical studies, the use of CD20 antibodies to deplete tumor-infiltrating B cells displayed different effects on tumor progression. In fact, the prognostic study or depletion of the entire population of tumor-infiltrating B cells in pan-cancer can be compared to that of T cells. This suggests the need to conduct an in-depth exploration of the role of B cell subsets in tumors. Tumor-infiltrating B cells have been found to be associated with a better prognosis in NPC 93, head and neck squamous cell carcinoma (HNSCC) 94,95, breast cancer 96, gastric cancer 97, HCC, CRC 98, ovarian cancer 99, prostate cancer 100, and melanoma 101 (Table 3). This manifests as improved prognostic indicators such as OS, Disease-Free Survival (DFS), Disease-Specific Survival (DSS), and reduced risk of recurrence. However, high densities of CD8^+^ T cells and DC-LAMP^+^ DC cells are correlated with shorter OS in RCC patients and lung metastasis 101,102. In lung adenocarcinoma patients with a high plasma cell gene score, there is less infiltration of B cells, CD8^+^ T cells, CD4^+^ T cells, and dendritic cells, indicating a weak immune response and a poor prognosis. Notably, high density infiltration and positive prognostic effects of B cells (except for Breg) may be highly dependent on mature TLS formation 24.

The impact of TLS on the control of tumor development and metastasis has been widely reported. In HNSCC 103, lung cancer, breast cancer 104, gastric cancer 105, HCC 106, pancreatic cancer 107, and CRC 108, urothelial carcinoma 109, the TLS signature is associated with prolonged OS and DFS in patients. These results may be partly attributed to the fact that HEV and TLS facilitate the infiltration of circulating immune cells into tumors and initiate local immune responses. Indeed, HEV, 12-CS, and DC-LAMP, which serve as components of TLS, are frequently used as alternative indicators of tumor prognosis and have positive effects on a variety of tumors 110. A multicenter propensity score-matched study indicated that patients with a high TLS density in right-sided colon cancer have a better prognosis than those with a low TLS density 108. Another relevant study revealed that the distribution and abundance of TLS were associated with molecular subtypes and clinical outcomes in patients with colorectal cancer liver metastases but not in those with pulmonary metastases 111. Additionally, mature TLS are believed to be associated with a reduced risk of early recurrence in hepatocellular carcinoma 112. The location of TLS also shows differences in tumor prognosis, with TLS located farther away from the tumor being associated with poor prognosis, whereas TLS around the tumor predicts a favorable prognosis 113. Nevertheless, TLS suppressive components, including TLS-associated Tregs and tumor cells detected are often associated with tumor progression and advanced stages 114. Overall, one plausible explanation of the results is that TLS and TIBs can provide sites for T cell activation and additional antigen stimulation, which is beneficial for anti-tumor immunity and patient prognosis.

### The predictive value of TIBs and TLS in response to cancer immunotherapy

Recent studies have shown that TIBs and TLS exert beneficial effects in predicting the clinical outcomes of cancer patients undergoing immunotherapy. It should be noted that on the one hand, TLS can serve as a predictor of the ICB response in tumor patients. On the other hand, neoadjuvant ICB treatment, radiotherapy, and chemotherapy can further induce TLS formation.

Lin *et al.* showed that CXCL13 secreted by tumor-infiltrating immune cells is significantly correlated with the immune responses to cancer immunotherapy in HNSCC 115. However, IL-35 was reported as a negative indicator in antitumor immunity and response to immunotherapy in PDAC 116. TLS-enriched type has a better prognosis and response to ICB therapy. The proximity of TLSs to tumor was found to be a key indicator of ICB response in HNSCC, while patients with TLS located far from tumor cells had a poorer prognosis 117. Similar results were observed in lung squamous cell carcinoma. In advanced NSCLC, TLS has been shown to predict the response to ICB independently of PD-L1 expression and CD8^+^ T cell density, but not the outcome of chemotherapy 118. In contrast to NSCLC, the density of follicular helper T cells in high-grade serous ovarian cancer (HGSOC) is not adequate enough to initiate functional B cell and mature TLS formation, which may be insufficient to maintain the ICB-sensitive TCF1^+^PD-1^+^CD8^+^ T cell phenotype 119. In addition, the presence of TLS and Trm cells in tissues is conducive to a better response in gastric cancer patients undergoing anti-PD-1 therapy 120.

In human LUAD, tumor-binding antibodies against endogenous retroviruses (ERV) exert antitumor activity and ERV expression predicts the outcome of ICB therapy 121. In patients with advanced TNBC responding to paclitaxel or its combination with the anti-PD-L1 atezolizumab, researchers found that follicular B cells and conventional type 1 DCs are considered as the TLS organizers and consistently increased after combination therapy 122. In another clinical study on the spatial predictors of the immunotherapy response to TNBC, immune interactions between B cells and granzyme B^+^T cells were shown to be secondary predictive molecules after CD8^+^TCF^+^T cells 123. A recent letter described the ability of TLS to predict the response to neoadjuvant chemotherapy and benefit from immunotherapy in HER2-negative breast cancer 124. Helmink *et al.* assessed the favorable role of TIBs and TLS in response to immunotherapy with ICB treatment through bulk and single-cell RNA sequencing in patients with melanoma and RCC 101. Similarly, in patients with urothelial carcinoma, high densities of B cells, T cells and TLS could predict the response to combined PD-1 and CTLA4 blockade treatment 125. In a phase 2 clinical trial of soft-tissue sarcoma, B cells remained the strongest prognostic factor, even in the context of low CD8^+^ T cells and cytotoxic content. The class E, which is abundant in TLS and TIBs, has shown improved prognosis and a high response to PD-1 blockade with pembrolizumab 29. Italiano *et al.* have revealed that the presence of TLS in advanced soft-tissue sarcoma is a potential predictive biomarker for improved patient response to pembrolizumab treatment 126. These favorable results of immunotherapy are closely related to the high expression of inhibitory molecules in the TME and the functions of TLS and TIBs in tumors.

## Insights into mechanism research of B cell in pan-cancer

### Glioblastoma

**Figure 4:**
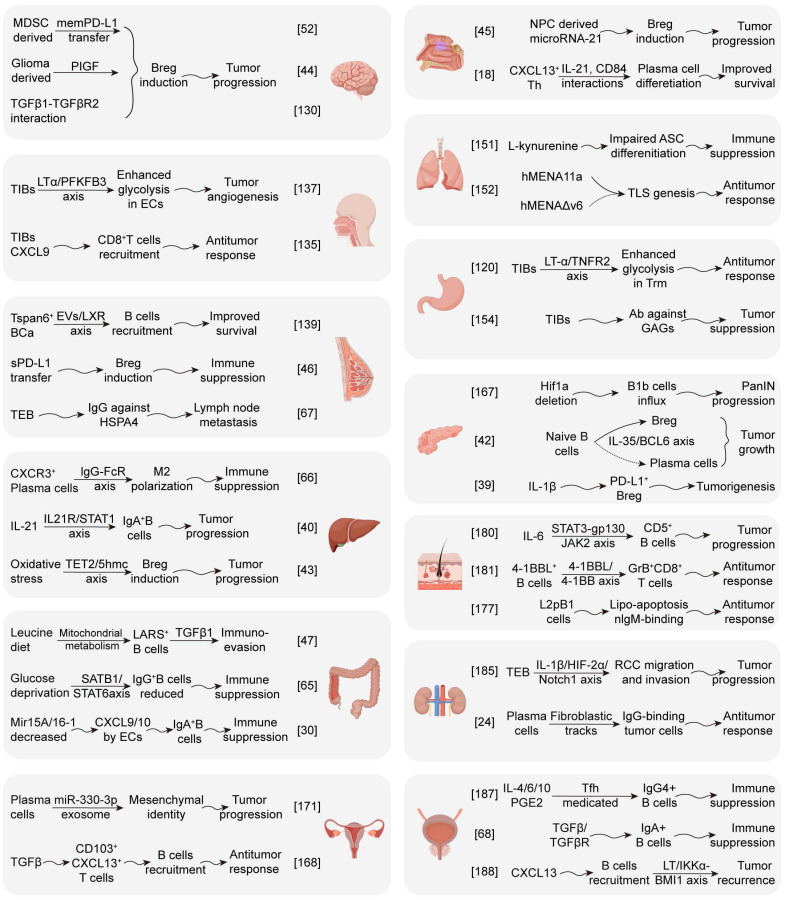
Overview of partial mechanism research on B cells and subsets in the pan-cancer field. The recruitment, differentiation, and effector functions of TIB subsets significantly impact immune response and tumor progression. Solid lines indicate promotion, and dashed lines indicate inhibition (By Figdraw).

Glioblastoma (GBM) is the most common malignant primary brain tumor in adults. ICB therapy has revolutionized cancer treatment; however, GBM patients have yet to benefit from this breakthrough. The primary obstacle in finding effective immunotherapy strategies is partially attributed to the low infiltration rate of TILs and a shortage of appropriate lymphatic vessels within the brain parenchyma 127. A study of tumor infiltrating immune cell profiles revealed the lymphocyte proportion in GBM, primarily including CD4^+^ T (0.5%±0.7%) and CD8^+^ T cells (0.6% ± 0.7%), and smaller numbers of CD4^-^CD8^-^T cells (0.2% ± 0.4%), Tregs (0.1% ± 0.2%), B lymphocytes (0.1% ± 0.2%), and NK cells (0.05% ± 0.05 %) 128. B cells and T cells are typically clustered within a radius of 15μm, while this proximity leads to the formation of immune synapses within the TME of GBM, mediating the inhibition of CD8^+^ T cell activation 129. In addition, GBM-associated MDSCs could promote the regulatory function of B cells through delivering membrane-bound PD-L1, endowing Bregs with the potential to inhibit CD8^+^ T cell activation and acquisition of the effector function 52. It has been reported that glioma cell-derived placental growth factor (PlGF) is also involved in the generation of regulatory B cells 44. Another study revealed that the TGFβ-1/TGFβR2 interaction is identified as the key molecule in the regulatory functions of tumor-infiltrating B cells in GBM 130. Furthermore, researchers have attempted to alter the regulatory effect of B cells through stimulating B cells with CD40 agonists, IFN-γ and BAFF to enhance the antigen presentation function of TIBs, thus facilitating antitumor response in GBM 129. In general, analysis of resection specimens from IDH1/2 wild-type GBM has shown that the association between CD20^+^Ki67^+^ B cells and prolonged survival is not significant. Furthermore, GBM contains focal B and T cell aggregates that resemble immature TLS 131. Although the presence of TLS assembled within brain tumors may serve as supplementary sites for antigen presentation and T cell initiation, the efficiency of tumor immune cycle in the central nervous system is weaker than that in the peripheral nervous system (Figure 4).

### Nasopharyngeal carcinoma

Nasopharyngeal carcinoma is an epithelial cancer originating from the mucosal layer of the nasopharynx and is characterized by Epstein-Barr virus (EBV) infection and regional epidemiology. The intense immune infiltration with poor differentiation of NPC is a unique feature that significantly distinguishes NPC from other cancers 132. Latent EBV infection is typically established in host B lymphocytes and epithelial cells, resulting in an increase in B-cell clonality. Importantly, significant and positive associations are observed between patient survival and the signatures of plasma cells in NPC cohorts 93. Li *et al.* demonstrated that TIBs recruited by CXCL13^+^ Th cells could induce plasma cell differentiation and anti-tumor immunoglobulin production through IL-21 and CD84 interactions in TLS 18. Notably, elevated serum IgA levels indicate continuous exposure to EBV and serve as high-risk biomarkers of NPC, whereas elevated titers of EBV neutralizing antibodies and anti-gp350 antibodies are considered low-risk biomarkers for NPC. These data suggest that vaccines targeting EBV-gp350 may reduce the risk of NPC. The identification of MEF2B, EBF1, and IL-6R as direct gene targets of EBV nuclear antigen 1 is critical for the survival of B lymphocytes in NPC 133. Nevertheless, researchers have found that NPC-derived miR-21 induces the formation of IL-10^+^ B cells, capable of suppressing the activities of CD8^+^ T cells. Therefore, miR-21 may represent a potential target for the treatment of NPC 45. In addition, there are studies showing that DNBs participate in regulating immune responses and shaping a suppressive TME in NPC 134. For further research on plasma cells, Bregs, and DNBs within the TME may reveal a broader role for B cells in NPC.

### Squamous cell carcinoma of head and neck

Head and neck squamous cell carcinoma refers to malignant squamous epithelial tumors, that typically arise from exposure to environmental carcinogens or human papillomavirus (HPV) infections. In oropharyngeal squamous cell carcinoma, TIBs may recruit CD8^+^ T cells through CXCL9 and high-density CD20^+^ B-cell/CD8^+^ T-cell aggregation is correlated with good prognosis in patients 135. In HPV^+^ HNSCC, researchers have revealed that TIBs contain a high proportion of activated, antigen-presenting, and memory B cell characteristics through flow cytometry. Patients have transcriptional signatures of GC BCs and spatial organization of immune cells consistent with TLS with GCs, both of which correlate with favorable outcome 94,95. The presence of tumor-specific B cell responses is marked by ASCs in the tumor stroma, which actively synthesize specific IgG antibodies against the HPV proteins E2, E6, and E7 *in situ*. Studies have shown that LT-α can promote tumor-associated TLS formation, and potentiate recruitment of CD8^+^ T cells and anti-tumor effects in HNSCC 136.

Although TLS and B cells show positive prognostic effects in HNSCC patients, the functions of regulatory B cell subsets have also been revealed by some researchers. Yang *et al.* reported that LT-α secreted by TILs promotes proliferation, migration and angiogenesis of endothelial cells by enhancing TNFR/NF-κB/PFKFB3-mediated glycolysis, which may lead to aberrant tumor angiogenesis and progression in HNSCC 137. In addition, multiple immunosuppressive mechanisms of regulatory B cells have been identified through ADO production in the TME, inhibiting T cell function 138. The inhibitory effect of ibrutinib on Bruton's tyrosine kinase (BTK) reduces the ADO production by downregulating CD39, significantly increasing B cell infiltration and impede tumor progression in HNSCC.

### Breast cancer

Breast cancer, which can be divided into hormone receptor-positive breast cancer, human epidermal growth factor receptor 2-positive breast cancer and triple-negative breast cancer (TNBC), is the most common malignancy among women worldwide. TIBs can sustain a humoral immune response in breast cancer and help generate effective anti-tumor immunity at the tumor situ. It is found that B lymphocytes could be recruited by tumor-derived extracellular vesicles (CCD-EVs) in a liver X receptor-tetraspanin 6 (LXR-Tspan6) dependent manner 139. A single-cell landscape of breast cancer has shown that intratumoral B cells are primarily naïve or memory cells, rather than ASCs 140. Compared with peripheral blood, they are more mature, have greater clonality, and greater CSR and SHM characteristics 96. Using immune receptor sequencing and RNA-seq, researchers found that B cell and T cell responses appear to coevolve with metastatic cancer genomes and reflect tumor mutations and neoantigen structures in breast cancer 141.

Notably, serum-derived soluble PD-L1 can facilitate PD-1^+^ regulatory B cells induction and IL-10 secretion, thus mediating the immune suppression in invasive breast cancer 46. Several studies have been conducted on the progression of TDLNs in breast cancer. Tacconi and his colleagues revealed that CD169^+^ macrophages, the predominant subtype in naïve lymph nodes, exert anti-metastatic effects with the presence of B cells 142. However, tumor-educated B cells (TEB) accumulate in TDLNs and selectively promote breast cancer lymph node metastasis by producing pathogenic IgG against HSPA4 67. In patients receiving neoadjuvant therapy, a positive feedback loop between activated CD24hiCD27^+^ Bregs and residual tumor cells in the TDLNs promotes multidrug resistance in breast cancer cells 143. Another study demonstrated that TNFhiIL10^+^ B cells in TDLNs showed an inverse correlation with the involved lymph nodes, indicating good clinical outcomes in patients with breast cancer 144.

### Lung cancer

Lung cancer, including non-small cell lung cancer and small cell lung cancer, is one of the most commonly diagnosed cancers and the leading cause of cancer-related death worldwide. Indeed, naïve-like and plasma-like B cells dominate the TME of lung cancer. A high plasma cell infiltration often signifies a poorer prognosis for LUAD patients 118, whereas it demonstrates greater sensitivity to drug treatments 145. IHC evaluation of LUAD tissue samples revealed that B cells and plasma cells were observed almost exclusively in the invasive tissue stroma 146. Similarly, another analysis of lung cancer tissue revealed that intratumoral B cells and plasma cells were mainly localized in the tumor TLS 147. Naïve-like B cells inhibit the growth of lung cancer cells by secreting molecules that negatively regulate cell growth, whereas plasma-like B cells inhibit cancer cell growth in the early stages of NSCLC but promote cell growth in the advanced stages of NSCLC 148.

In addition, some studies have shown that high frequencies of CD24^hi^IL-10^+^ Bregs with plasmacytoid gene signatures are found in lung cancer patients 149. CD27^-^lgD^-^double-negative B cells in NSCLC have also been identified and shown a negative correlation with the presence of mature B cell populations 150. In flamed NSCLC, IDO1 is upregulated in cancer cells and TLS, increasing tryptophan degradation and metabolite kynurenine production, thus mediating an immunosuppressive effect 151. Moreover, a study by Modugno *et al.* indicated that NSCLC tumoral hMENA11a upregulated LTβR, decreased fibronectin, and favored CXCL13 production and TLS formation by Trms. In contrast, hMENAΔv6 in cancer-associated fibroblasts inhibited LTβR-associated TLS formation via NF-kB pathway 152.

### Gastric cancer

Gastric cancer is the fifth most common cancer and the third leading cause of cancer death worldwide. High levels of CD20^+^ B cell infiltration are significantly associated with improvements in overall survival and disease-free survival of GC patients 97. Most infiltrating B cells in gastric cancer around the tumor margin in the form of TLS are sensitized by antigens and serve as APCs in TLS, participating in the induction of cytotoxic T cells 153. Researchers have revealed that sulfated glycosaminoglycans (GAGs) are the major functional B cell antigens in gastric tumors. Utilizing natural anti-sulfated GAG antibodies inhibits the growth of various human malignancies 154. Another study indicated that CD103^+^ T cells in the tumor epithelium were located around TLS and CD103^+^CD8^+^ Trm cells in tumor were associated with the TLS, leading to the enhancement of anti-tumor immunity in gastric cancer 155. TLS associated B cells could promote glycolysis of CD103^+^CD8^+^Trm cells through the LT-α/TNFR2 axis, enhancing the secretion of CXCL13, granzyme B and antitumor immunity in gastric cancer tissues 120.

In addition, Hu *et al.* found that IL-10-expressing CD27^+^CD10^-^ B cells aggregate in the intratumoral environment of gastric cancer, which may significantly reduce the production of IFN-γ, TNF, and IL-17 and inhibit antitumor response 156. Similarly, CD19^+^CD24^hi^CD27^+^Bregs could inhibit IFN-γ production of CD4^+^T cells and promote immune escape in gastric cancer 157. Furthermore, it is reported that Bregs in the cancerous mucosa and PBMC increase with cancer stage and promote gastric carcinogenesis by inducing the production of inflammatory mediators IL-10 34. With the levels of Tfh, Breg, and CXCL13 increased, related lymph node metastasis and poor prognosis have been observed in gastric cancer patients.

### Liver cancer

Liver cancer remains a global health challenge, with hepatocellular carcinoma as the most common form. In the past decade, several studies have been conducted on functional B cells and Breg subsets of TIBs in HCC. The close proximity of TILs and B cells is associated with heightened intratumor immunity and contribute to an improved prognosis for patients with HCC 75. Importantly, the refined immunosubtype, derived from the spatial dynamics of T and B cell responses, can effectively predict the clinical trajectory following surgical resection of resectable HCC and Atezo/Bev therapy for advanced HCC 158. In addition, the immunogenic subtype of intrahepatic cholangiocarcinoma is characterized by inflammatory infiltration and is associated with prolonged patient survival, featuring a variety of immune cells, including effector and memory T cells, B cells, and macrophages 159. Intra-tumoral TLS are linked to a reduced risk of early relapse following surgical resection and effective *in situ* anti-tumor immunity 112. These results suggest that local immunity is activated in the early stage of HCC.

Researchers uncovered that CXCR3^+^ B cells account for approximately 45% of TIBs and related to early recurrence of human HCC 16. The crosstalk elucidated between macrophages and CXCR3^+^IgG^+^ plasma could promote inhibitory cytokines secretion of macrophages and diminish the effect of CD8^+^ T cells in the murine models of HCC 66. Furthermore, it has been reported that the IL-21R-STAT1 axis is activated in MASH-driven HCC, leading to the induction of immunosuppressive IgA^+^ plasma cells and tumor progression 40. Several intervention strategies involving Breg have been reported. Inhibiting the catalytic activity of ten-eleven translocation-2 (TET2) enzymes in B cells can enhance antitumor immunity and improve the efficacy of PD-1 therapy in HCC 43. In particular, it has been suggested that the ablation of B cells by CD20 antibodies can limit hepatic fibrosis-driven tumorigenesis in HCC mouse models. Besides, high infiltration of FcγRII^low/-^ B cells activated by semimature DCs is linked to cancer progression and poor prognosis, whereas the interaction between tumor-infiltrating T cells and B cells has also been identified and improved outcomes in HCC patients 75,84.

### Colorectal cancer

In recent years, the incidence and mortality rates of CRC have increased globally. Mature TLS and plasma cells have been reported in CRC or adjacent tissue, which are associated with improved clinical outcomes in patients. In colorectal cancer liver metastasis, CCL19^+^ fibroblasts have been found to facilitate lymphocyte recruitment to TLS, thereby enhancing the antitumor immunity of IgG^+^ plasma cells 98. CD86^+^ antigen-presenting B cells were identified in the GC of TLS and isolated from TDLNs, inducing responses from autologous T cells *in vitro* 160. Human IL-36γ was associated with CD4^+^ central memory T cell infiltration, increased B cell density in TLS, and markers of fibrosis, supporting IL-36γ as the physiological immune responses through maintaining inflammation in CRC 161. It is reported that declined micro15A/16-1 levels in tumors increased I-kappaB kinase-mediated NF-κB/STAT1 activation, which lead to the production of chemokines CXCL9 and CXCL10 of epithelial cells and promote the progression of CRC through chemotaxis of IgA^+^IL-10^+^ plasma cells 30. Wang *et al.* demonstrated that LARS2^+^ B cell subset shows a regulatory phenotype and is located outside the TLS, while leucine starvation could inhibit LARS2 B cell-mediated immune evasion in CRC 47. Moreover, researchers have revealed that the deceleration of glucose metabolism in TIBs suppresses IgG^+^ plasma cell differentiation through the SATB1 pathway, whereas intervention in glucose metabolism may enhance the anti-tumor effect of B cells 65.

### Pancreatic cancer

Pancreatic cancer has the worst prognosis among all common solid malignancies, with a 5-year overall survival rate of approximately 10%. Exocrine pancreatic cancer accounts for approximately 95% of all pancreatic cancer cases, with the most common type being ductal adenocarcinoma. PDAC exhibits a highly suppressive TME and significant B cell infiltration, characterized by extensive inflammatory fibrosis, desmoplastic stromal reaction, and hypo-vascularity 162. Further analysis of PDAC tissue shows that groups with mature TLS support plasma cell differentiation as well as the formation of tumor-reactive T cells 163. In the peripheral blood of PDAC patients, circulating plasmablasts proliferate and infiltrate pancreatic lesions, stimulating collagen production in fibroblasts and participating in extracellular matrix remodeling 28. Autoantibodies targeting filamentous actin and nuclear protein RuvB-like AAA ATPase2 antigens have been detected in the TME and peripheral blood, supporting the initiation of humoral immunity in the chronic inflammatory microenvironment of PDAC 164. Capello *et al.* showed that tumor antigens released by exosomes from PDAC could trigger the production of autoreactive antibodies and serve as decoys for complement-mediated cytotoxicity 165.

In addition, various phenotypes of IL-35 producing Bregs including CD1d^hi^CD5^+^ B cells and CD38^+^ B cells have been reported in PDAC 37,54,166. Studies have shown that IL-1β of TME promotes tumor progression through increasing CXCL13 expression and infiltration of immune-suppressive IL-35^+^ B cells 39. Dysregulated transcriptional programs resulting from IL-35-induced BCL6 upregulation in B cells, impair the differentiation of naïve B cells into antitumor plasma cells through the stimulation of STAT3-PAX5 complex in PDAC 42. For B-cell-mediated IL-35 induction, BCR stimulation of BTK signaling is necessary and inhibition of the key downstream molecule PDK2 enhances effector T cell function 54,55. Researchers have uncovered that IL-35 production by circulating B cells in PDAC indicates CD8^+^ T cell exclusion and immunotherapy resistance via IL-35/gp130/STAT3 35. Furthermore, hypoxia-inducible factor 1α (HIF1α) has been identified as a protective factor in the preinvasive phase of PDAC, since its absence causes pro-tumor B1b influx and tumor progression 167.

### Ovarian cancer

Ovarian cancer is the second most common cause of gynecological cancer-related death among women worldwide. Analysis of tumors and stromal cells reveals that intratumoral plasma cells are associated with a better prognosis in patients with HGSOC 99. Ovarian clear-cell carcinomas with an inflammatory stroma constitute a unique clinicopathological subgroup, and these tumor cells induce inflammation and stimulate plasma cell differentiation in a paracrine manner. TGFβR signaling licensed CD8^+^ T cells upregulate CD103 and secrete CXCL13 with TCR stimulation, involved in mediating B cell recruitment and TLS formation in ovarian cancer 168. Studies have pointed out that IgA-binding tumor antigens can mediate the transcytosis and cytosis, promoting cytotoxic T lymphocyte (CTL) killing of tumor cells and inhibiting tumor growth in ovarian cancer through myeloid cell-dependent mechanisms, neutralizing secreted factors, or poly-Ig receptors on cancer cells 60. Intratumoral ASCs also produce tumor-reactive IgG targeting MMP14 in HGSOC, specifically encapsulating tumor cells with high MMP14 expression 169. In omental metastases of HGSOC, B cells primarily infiltrate lymphoid structures in the stroma. Tumor-specific IgG is produced and mediates a strong B cell memory response against a limited antigen repertoire, supporting antitumor response 170. T cells and B cells colocalized in lymphoid aggregates, ranging from small diffuse clusters to well-organized TLS, suggesting that synergistic interactions could generate and enhance the effective antitumor immunity in HGSOC 74.

However, Yang *et al.* found that the switch of HGSOC-mesenchymal phenotype can be triggered by the transportation of plasma cell-derived exosomes containing miR-330-3p, which increases the expression of junctional adhesion molecule B in an atypical manner and is associated with tumor development 171. In addition, Bregs has been reported to preferentially enrich ascites in ovarian cancer and impair anti-tumor immunity 172. In the ovarian cancer murine model, peritoneal ascite-B1 cells, spleen B1 cells and marginal zone B cell subsets were selectively lost due to the influx of MDSCs 173. In fact, mature TLS are formed only in 16% of HGSOC with a relatively high TMB 119. It is well-supported that the anti-tumor immune effects of humoral immunity and tumor-associated TLS in ovarian cancer have not been fully elucidated.

### Melanoma

Cutaneous melanoma is a malignant tumor that arises from melanocytes in the skin. Current evidence supports that B cells enhance tumor immunity against B16F10 melanoma by promoting T cell infiltration into tumors and cytokine production in the TME, associated with better prognosis in melanoma 174. When exposed to melanoma secretomes, B cells express inflammatory factors, T cell-recruiting chemokines, differentiating into plasmablast-like cells to sustain inflammatory state 175. The co-occurrence of tumor-associated CD8^+^ T cells and CD20^+^ B cells is associated with improved survival, independently of other clinical variables 176. In addition, L2pB1 cells, as a subpopulation of B1b cells, have been reported to inhibit melanoma growth by inducing lipo-apoptosis of tumor cells 177. Immature and follicular B2 cells exert protective effects against melanoma progression by promoting the generation of effector memory T cells 178. As the macrophage barrier is disrupted with the application of therapeutic agents, the contact of tumor-derived vesicles with B cells can enhance tumor-related humoral immunity in TDLNs 82.

Extensive findings underscore the diverse roles of B cells in the peripheral blood, TME, and TDLNs of melanoma. In addition, IL-10^+^ B1a cells selectively accumulate in melanoma and promote tumor growth by inhibiting tumor-infiltrating CD8^+^ T cells 38. In TDLNs of B16F10 melanoma, researchers have identified a subset of TIM-1^+^ B cells, while selective deletion of TIM-1 enhances the IFN-γ response and anti-tumor immunity 179. Animal experiments have shown that IL-6 binds to CD5^+^ B cells and can activate the transcription factor STAT3 through gp130 and its downstream kinase JAK2, thereby promoting the progression of melanoma 180. Lee-Chang* et al.* proposed that 4-1BBL^+^ B cells could increase antitumor responses in aged mice by presenting endogenous antigens and inducing GrB^+^CD8^+^ T cells via the 4-1BBL/4-1BB axis 181.

### Renal clear cell carcinoma

Renal cell carcinoma (RCC) is the most common form of kidney cancer and is typically divided into clear cell carcinoma (ccRCC) and non-clear cell renal cell carcinoma. B cell repertoire analysis revealed clonal diversification, selection, and expansion of B cells, along with the prolonged PFS and response to ICI in TLS^+^ ccRCC. Further studies have shown that IgG- and IgA-producing B cells spread into the tumor bed along fibroblast tracks in TLS 24. Analysis of genomic alterations in TLS shows that the PI3K-mTOR pathway and the proportion of PD-1^+^ cells are generally upregulated in TLS^+^ tumors of ccRCC 182. Xu *et al.* showed that most TLS in ccRCC are located in the distal areas of the tumor and are associated with immature and immunosuppressive characteristics, whereas tumor-adjacent TLS correlate with favorable clinical outcomes 183. Similarly, another study also supported that the high density of intratumoral TLS is an independent risk factor for a favorable prognosis in ccRCC 184. Besides, it is reported that B cells recruited by tumor cells can activate IL-1β/HIF-2α signaling and induce the downstream Notch1 signaling pathway, significantly increasing the migration and invasion of RCC cells 185.

### Prostate cancer

Prostate cancer progresses from prostatic intraepithelial neoplasia to locally invasive adenocarcinoma, and eventually to castration-resistant metastatic cancer. Prostate tumors in Black men of African descent exhibit enhanced plasma cell infiltration, elevated markers of NK cell activity, and increased IgG expression, which are associated with better outcomes 100. Studies have shown that B cell infiltration is higher in intratumoral areas than the peritumoral areas of prostate cancer tissues during prostatectomy 186. The proportion of Tfh2 and IgG4^+^ B cells in patients with prostate cancer is increased compared with healthy peripheral blood. Further studies have shown that cytokines, such as IL-4, IL-6, IL-10 and PGE2, can promote the antibody class switching of B cells to IgG4, partly through the induction of Tfh2 cells, which is related to the immunosuppressive microenvironment 187. In addition, it has been found that the CXCL13 recruits B cells into prostate cancer tumors and activate the IκB kinase α-BMI1 module in cancer stem cells through the production of LT, thus promoting the progression of castration-resistant prostate cancer 188. Shalapour *et al.* identified the key TGFβR signaling immunosuppressive B cells in prostate cancer as plasma cells expressing IgA, IL-10, and PD-L1, which are involved in tumor immune tolerance 68.

## Prospects of B cells and TLS in tumor immunotherapy

Immunotherapy has become a new pillar of cancer treatment through blocking receptors and ligands that weaken T cell activation pathways, reversing the state of T cell exhaustion in the TME. Drug resistance and immune-related adverse events (IRAEs) limit the further application of ICB. There is evidence that B cells and antibodies can serve as predictors of the IRAEs during ICB therapy 189. Given the observed link between TLS and clinical outcomes in various cancer, targeting B cells and manipulating TLS may present an appealing therapeutic approach.

### B cells and antibodies-based tumor treatment strategies

Preclinical research and clinical applications focuses on B cell treatment strategies including depletion of Breg, anti-tumor B cell activation, antibody production, cytokine therapy and B cell vaccines. B cell depletion can be used to treat autoimmune diseases, infections and malignant B cell diseases. Drugs for Breg depletion through CD20 antibodies pose risks and controversies in cancer treatment, given the association of B cells with a better prognosis in most tumors 190. Nano-capture technology of CXCL13 could reduce tBreg differentiation and normalize epithelial-mesenchymal transition in the TME, thereby inhibiting tumor growth and prolonging the PFS in pancreatic cancer, BRAF mutant melanoma, and TNBC murine models 191. Intratumoral delivery of CCL21 nanoparticles has been associated with the inhibition of lung cancer growth, reflecting the complexity of chemokine-based therapies 192. In addition, it is reported that nano-delivery therapy of PI3K-γ inhibitors enhance the anti-tumor immunity by hampering cellular activation and migration to reduce suppressive myeloid and plasma cell infiltration in tumors 193. Inhibiting the BTK of B cell can restore T cell-dependent anti-tumor responses and enhance sensitivity to chemotherapy treatments 194. The BTK inhibitor Ibrutinib impedes PDAC progression and improves response to chemotherapy, providing a new treatment for pancreatic cancer.

**Figure 5:**
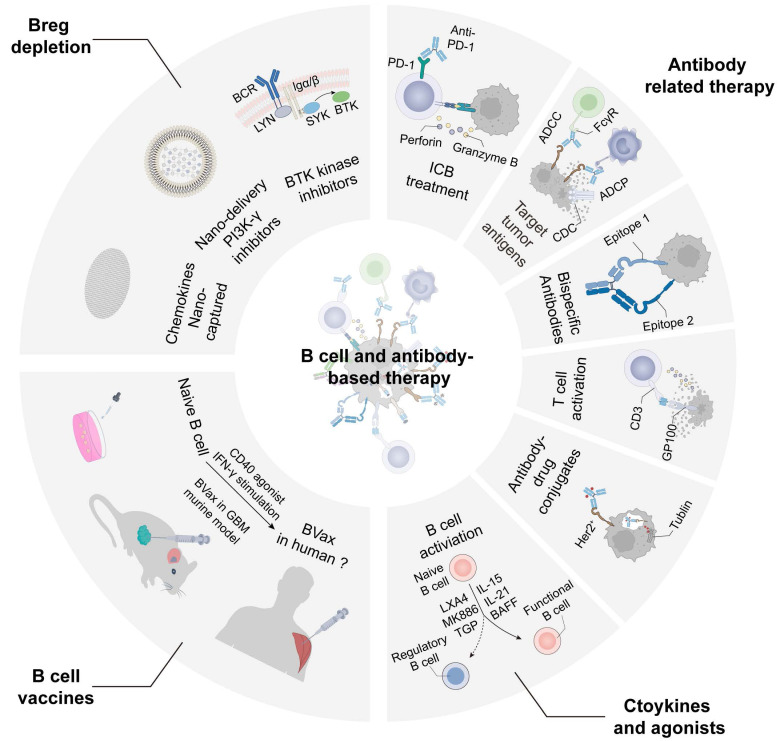
B cell and antibody-based therapeutic strategies. Current B cell therapy in cancer is primarily utilized in preclinical stages within *in vitro* and animal models, whereas engineered antibodies have been widely adopted in clinical applications due to their antigen-specificity. TGP: total glucosides of paeony; LXA4: lipoxin A4.

Engineered antibodies have significantly expanded the strategic spectrum of targeted therapy and immunotherapy, providing promising perspectives for cancer treatment 195. Monoclonal antibodies are developed for ICB therapies, anti-angiogenic drugs, antibodies targeting tumor antigens, and antibody-drug conjugates incorporating drugs, radioisotopes and immunotoxins, while bispecific antibodies are also employed in cancer treatment through targeting T cells plus tumor epitopes or double tumor antigen epitopes (Figure 5). There are various studies reported B cell activation components (IL-2, IL-6, IL-15, IL-21, BAFF, APRIL, rhIL-12, fucose and CXCL13-coupled CpG-ODN) and Breg suppressive agents (lipoxin A4, MK886, total glucosides of paeony and resveratrol) to regulate the proportion of functional B cells in TME 49. Under the concept of therapeutic cancer vaccines, activation of 4-1BBL^+^ B cell vaccines with CD40 agonism and IFN-γ elicits potent immunity effects in GBM murine models by enhancing antigen-presenting and antibody-secretion capabilities through *in vitro* stimulation and reinfusion 129. These studies highlight the potential applications of B cells and antibodies for cancer treatment, emphasizing the need to enhance anti-tumor B cells and antibodies while concurrently inhibiting the Breg phenotype.

### TLS-based tumor treatment strategies

Based on the positive impact of TLS in the prognosis of tumor patients, devising methods to induce TLS formation has become an effective strategy. Several studies have shown that enhancing tumor-related TLS can broaden the scope of current cancer treatment in various systemic and intratumoral manners. Among systemic therapies, approaches such as radiotherapy 196 and chemotherapy 197 in mice and human have shown the potential to induce TLS, which are essential for maintaining the long-term responses to immunotherapy 198. While enhancing tumor immunity by increasing tumor-associated TLS is effective through systemic therapies, current intratumoral interventions need to specifically target and manipulate the TME. In murine models, it has been reported that increased levels of chemokines 199, cytokines (LT, TNFα, LIGHT-VTP, CXCL13/CCL21, IL-15, TGF-β, etc.) 200 and HEV have been reported to be involved in TLS genesis 110. The use of agonists to activate pathways that normalize tumor vasculature and enhance immune infiltration. Many studies have referred to the role of agonist agents 201. Oncolytic adenoviruses 202 and humanized cancer vaccines 203 serve as delivery systems for therapeutic agents to facilitate the genesis and function of TLS in tumors.

In addition, biomaterial materials including 3D scaffolds 204, engineered DCs 205, collagen matrices 206, hydrogels 207, nanomaterials 208 and organ-on-a-chip/organoids 209 have been developed to mimic and enhance immune structures within tumors. These approaches aim to establish a supportive environment for the formation of TLS, thereby increasing the capacity of the immune system to combat cancer. Collectively, these strategies exemplify multifaceted approaches to harnessing TLS in the pursuit of more effective cancer therapeutics (Table 4). Since TLS have the potential to induce the excessive activation of anti-tumor immunity *in situ*, it is necessary to consider the methods and risks that pan-specific manipulation of TLS may exacerbate IRAEs.

## Conclusions and perspectives

**Figure 6:**
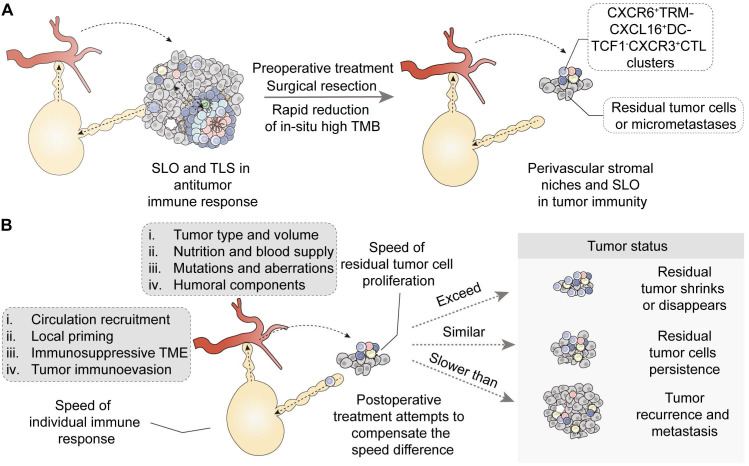
Postoperative models of individual immune response and tumor proliferation. (A) Surgical resection removes tumor tissue while also terminating the local immunity of intratumoral TLS. (B) Postoperative immunity targeting residual tumor cells requires the reactivation of circulating activation pathways. The hysteresis in circulating immunity and the re-establishment of local immunity provide the opportunity for rapid tumor growth. The speed difference of the individual immune response and tumor growth determine the status of tumor development.

Particularly, surgical resection, alleviating the immunosuppressive TME, helps achieve an equal fight between immune cells and residual tumor cells. Postoperative immunotherapy and targeted therapy can support the individual immune system and effectively eliminate residual tumor cells and micrometastases. However, postoperative drug resistance, along with tumor recurrence and metastasis, continues to pose significant challenges in the treatment of solid tumors. It should be pointed out that surgical treatment removes tumor tissue but also terminates the intratumoral TLS effect. Although CXCR6^+^ tissue-resident memory T and B cell clones clustering with CXCL16^+^ DCs allows the survival of transitory effector state TCF1^-^CX3CR1^+^ CTLs, they may be not adequate for response to tumor recurrence and still require additional circulating recruitment 15. In essence, tumor recurrence and metastasis can be interpreted as the insufficiency of local priming and the hysteresis of circulating immunity. The time difference between the individual response to tumor specific antigens and the proliferation of residual tumor cells determines the possibility and risk of recurrence after surgery, which is analogous to the tortoise and the hare race. It is particularly important to establish effective local immunity and immunological memory to eradicate remaining tumor cells through biocompatible engineered materials after surgery. Therefore, utilizing engineered antibodies, therapeutic tumor vaccines and establishing biocompatible mature TLS may potentially make up for this time difference and provide a higher response likelihood for tumor antigens that lymphocytes encounter (Figure 6). Future treatments need to explore effective strategies for cancer treatment from multiple dimensions including intensity and speed to attain more therapeutic benefits.

## Figures and Tables

**Table 1:** B cell activation pathways in cancer immunity cycle

Feature	Humoral Circulation Activation Pathway	Intratumoral Cycle Activation Pathway
Activation Location	Secondary lymphoid organs	Tumor-induced TLS and LMAs
Activation& Response Path	Lymphatic and blood circulation	Recruitment from HEV; Response along fibroblastic tracks
Activation Manners	Lymph nodes: GC and EF; Spleen: marginal zone	GC pathway and EF pathway
Antigen Source	Circulating and draining tumor antigens	Intratumoral tumor antigens
Antigen Presentation	DCs, Macrophages	B cells offering the additional stimulus
Humoral immunity	B cells fully activated in peripheral lymphatic organs	Highly dependent on the distribution, density and maturity of tumor-associated TLS; Effective B cell clones fewer than TMB
Antibody Effect	Superficial layer of solid tumors, consistent with the vascular distribution	Inside the tumor derived from antibody secreting cells
Response Efficiency	Indirect, hysteretic but essential	Direct, efficient, but impaired
Relation	Initiation of intratumoral immunity	Extension and supplement of circulatory immunity trafficking into tumor core
Applications	Reconstitution of intratumoral immunity in the early postoperative period	In-situ response speed determining postoperative tumor status: tumor shrink, persistence, recurrence or metastasis

**Table 2:** Regulatory B cells identified in human and murine cancers

Cancer type	Surface marker	Location	Effector function	Refs
HNSCC	CD19^+^ CD19^+^CD73^+^CD39^+^CD23^+^CD21^-^	Human; PBMC Human; PBMC, Tumor	IL-10, PD-1 Adenosine	51 138
Breast carcer	CD19^+^ CD24^hi^CD27^+^	Human; Tumor Human; TDLN	Granzyme B IL-6, TNF-α	41 143
Gastric cancer	CD19^+^CD24^hi^CD38^hi^ CD19^+^CD24^hi^CD27^+^	Human; PBMC, Tumor Human; PBMC, Tumor	IL-10, TGF-β1 IL-10	34 157
HCC	CD19^+^CD5^hi^ CD27^hi^CD38^dim^ CD5^hi^CD24^-^CD27^-/+^CD38^+/hi^	Human; Tumor Human; PBMC, Tumor	PD-1, IL-10 IL-10	50 53
PDAC	CD19^+^CD24^hi^CD38^hi^ CD38^+^CD19^+^	Human; PBMC Human; Tumor	IL-35, IL-10 IL-10	35 166
CRC	CD19^lo^CD27^hi^ CD19^+^B220^-^CD20^-^IgD^-^CD38^+^CD27^+^ CD138^-^	Human; Tumor Human; Tumor	IL-10 TGF-β1	36 47
Ovarian carcinoma	CD19^+^CD20^+^ CD19^+^	Human; Ascites Human; Tumor	IL-10 Granzyme B	172 41
Cutaneous squamous cell carcinoma	CD19^+^CD73^-^CD25^+^CD71^+^TIM1^+^CD154^+^	Human; PBMC	IL-10	56
Breast cancer	CD19^+^CD81^hi^CD25^+^	Murine; Tumor	TGF-β1	49
HCC	CD19^+^ CD19^low^IgA^+^B220^-^CD138^+^ MHC-II^low^	Murine; Tumor Murine; Tumor	PD-L1, IL-10, Fas-L PD-L1, IL-10, TGF-β1	40 58
PDAC	CD19^+^CD1d^hi^CD5^+^ CD19^+^CD1d^hi^CD5^+^CD21^hi^ CD19^+^PD-L1^+^	Murine; Tumor Murine; Tumor Murine; Tumor	IL-35 IL-35 PD-L1	37 35,116 39
CRC	CD19^+^IgA^+^	Murine; Tumor	PD-L1, IL-10, TGF-β1	30
Prostate cancer	CD19^+^CD20^lo^B220^lo^IgA^+^PD-L1^+^	Murine; Tumor	IL-10	68
Melanoma	CD19^+^CD5^+^CD1d^int^CD43^+^	Murine; Tumor	IL-10	38

TDLN: tumor-draining lymph nodes; PBMC: peripheral blood mononuclear cell

**Table 3:** Depicting the prognostic and predictive value of TIBs and TLS in human cancers

Tumor type	Analysis factor	Detecting method	Prognostic Indication	Prognostic value	Predictive value	Refs
GBM	TIBs, density	IF	Patient survival	Neutral	NA	131
NPC	TIBs	RNA-seq	PFS	Favorable	NA	93
HNSCC	TLS TLS, TIBs TLS, location	RNA-seq, IF RNA-seq, IF Multi-omics	RFS PFS, OS, RFS NA	Favorable Favorable NA	NA NA Positive	103 95 117
Lung cancer	TLS Plasma cells Plasma cells	H&E, IHC RNA-seq RNA-seq	PFS, OS OS OS	NA NA Adverse	Positive Positive NA	147 118 145
Breast cancer	TIBs, density TLS TLS, density	RNA-seq 12-CS H&E, IHC	DFS, OS PCR OS, PFS	Favorable NA Favorable	NA Positive NA	96 124 104
Gastric cancer	TLS, mature TLS, Trm TIBs	H&E IF IHC	OS OS, DFS OS, DFS	Favorable NA Favorable	NA Positive NA	105 120 97
HCC	TLS Adjacent tumoral TLS	H&E H&E	RFS, OS Early/Late recurrence	Favorable Neutral	NA NA	106 112
PDAC	TLS, mature TLS, mature	RNA-seq, IF H&E, IHC	Survival probability OS, PFS	NA Favorable	Positive NA	163 107
CRC	TLS TLS, location, density TLS, plasma cells	IHC, H&E IHC H&E, IHC, 12-CS	OS OS, RFS RFS, OS	Favorable Favorable Favorable	NA NA NA	108 111 98
ccRCC	TLS, TIBs, IgG TLS	ST IHC	PFS OS	NA Adverse	Positive NA	24 102
Urothelial carcinoma	TLS TLS	IHC, H&E IHC	DFS OS, RFS	Favorable NA	NA Positive	109 125
Prostate cancer	Plasma cells	IHC	MFS, DFS	Favorable	NA	100
Ovarian cancer	Plasma cells	RNA-seq	OS	Favorable	NA	99
Melanoma	TLS, TIBs, density TLS, TIBs	IF, RNA-seq H&E, IHC	OS OS	NA NA	Positive Positive	101 176
Soft tissue sarcoma	TLS, TIBs TLS	IF, RNA-seq IHC	OS, PFS, ORR OS, PFS	NA NA	Positive Positive	29 126

IGKC: immunoglobulin kappa C; RFS: recurrence-free survival; FFS: failure-free survival; PFS: progression-free survival; MFS: metastasis-free survival.

**Table 4:** TLS induction strategies in mouse models and cancer patients

Categories	Treatment	Subject Type	Description	Refs
**Systemic therapies**	Radiotherapy	Mice	TLS with medullary breast cancer is described in a patient case report, and similar to TLS observed in the genetic mouse model.	196
	Chemotherapy	Patient	Chemotherapy induces C3 cleavage and CR2 activation, resulting in CR2^+^ B cells expressing ICOS-L, which accumulate in TLS.	197
	Immunotherapy	Patient	TLS may be involved in initiating and maintaining long-term responses to ICB.	101, 125, 147
	Targeted therapy: Light-VTP	Mice	Targeting LIGHT to tumor vessels via VTP leads to the formation of TLS and prolongs survival in mice when combined with ICB treatment.	198
**Intratumor therapies**	Chemokines: CXCL13/CXCR5 CCL21/CCR7	Mice	Under the control of the rat insulin, expression of LTα1β2 promoter induces HEV formation and secretion of CCL19/21, and CXCL13 in pancreatic tissue, leading to the development of TLS.	199
	Cytokines: Lymphotoxin-α Lymphotoxin-β	Mice	CD8^+^ T cells, together with TIBs that secrete lymphotoxin-α1β2 (LTα1β2), act as LTi cells and coordinate the formation and expansion of TLS.	200
	Agonistic agents: STING agonists TLR4 agonists CD40 agonists	Mice Mice Mice	Low doses of the STING agonist injection have been shown to promote tumor vascular normalization, increase immune infiltration, and induce the formation of immature TLS.	201
**Oncolytic virus**	Tumor-selective oncolytic virus vector	Mice	The identified 4-E03 antibody via oncolytic vaccinia vector exhibits stronger Treg depletion against Treg cells expressing CTLA-4, potentially enhancing the functionality of TLS.	202
**Humanized cancer vaccine**	(GM-CSF)-secreting PDAC cell vaccine (GVAX)	Patient	Compared with tumors from group of unvaccinated patients, patients vaccinated intradermally with irradiated GCAX induce an increase in TLS formation of tumor.	203
**Biomaterials**	3D TLS scaffold	Mice	The 3D scaffold with a porous structure can recruit numerous immune cells to endow it with functions similar to real lymphatic organs, thus forming an artificial tertiary lymphatic structure.	204
	Engineered DCs	Mice	T-bet and IL-36γ synergistically enhance each other's expression in DCs, enabling them to promote TLS formation in the “immune normalization” therapeutic TME.	205
	Collagen matrices	Mice	Transplantable and functional TLS are constructed by applying soluble factors, encapsulated within a slow-release gel.	206
	Hydrogels	Patient	By combining Zn2^+^ with 4,5-imidazole dicarboxylic acid and incorporating chitosan and CpG, a STING-activating hydrogel (ZCCG) is meticulously prepared to initiate and activate cGAS-STING and TLR9 pathway-mediated immunotherapy.	207
	Nanomaterials	Mice	A nano-vaccine, composed of EBNA1 and a bi-adjuvant combination of Mn2+ and CpG formulated with tannic acid, enhances the expression of downstream chemokines CCL19/21, and CXCL13 by activating the LT-α and LT-β pathways to promote the formation of TLS.	208
	Organ-on-a-Chip/organoids	Vitro	Primary B and T cells isolated from human blood are cultured in a 3D extracellular matrix gel and are able to independently assemble into ectopic lymphoid follicles.	209

VTP: vascular targeting peptide; T-bet: T-box expressed in T cells; CPG: cytosine phospho-guanine; EBNA1: Epstein-Barr virus nuclear antigen 1.

## Abbreviations

AHR: aryl hydrocarbon receptor
AtM B cell: atypical memory B cell
ADCC: antibody-dependent cellular cytotoxicity
ADO: adenosine
ADCP: antibody-dependent cellular phagocytosis
APC: antigen-presenting cell
ASC: antibody secreting cell
Breg: regulatory B cell
BAFF: B-cell activating factor
Breg: regulatory B cell
BTK: Bruton's tyrosine kinase
CCL21: CC-chemokine ligand 21
ccRCC: clear cell renal cell carcinoma
CDC: complement-dependent cytotoxicity
CCD-EV: cancer cell-derived extracellular vesicle
CLP: common lymphoid progenitor
CPG: cytosine phospho-guanine
CRC: colorectal cancer
CSR: class switch recombination
CTL: cytotoxic T lymphocyte
CXCL13: CXC-chemokine ligand 13
DFS: disease-free survival
DNB: double negative B cell
DSS: disease-specific survival
EBNA1: Epstein-Barr virus nuclear antigen 1
EBV: Epstein-Barr virus
EBF1: early B-cell factor 1
EF: extrafollicular
EF BC: extrafollicular B cell
ERV: endogenous retrovirus
FFS: failure-free survival
FDC: follicular dendritic cell
FRC: fibroblast reticular cell
FOXO1: Forkhead Box O1
GABA: γ-aminobutyric acid
GAG: glycosaminoglycan
GBM: glioblastoma
GC BC: germinal center B cell
GrB: granzyme B
H&E staining: hematoxylin and eosin staining
HA: hyaluronic acid
HCC: hepatocellular carcinoma
HGSOC: high-grade serous ovarian cancer
HEV: high endothelial vein
HIF1a: hypoxia-inducible factor 1α
HMGB1: high mobility group box 1
Hhep: helicobacter hepaticus
HNSCC: head and neck squamous cell carcinoma
HSC: hematopoietic stem cell
HSPA4: heat shock protein 4
ICB: immune checkpoint blockade
IDO: indoleamine-2,3-dioxygenase
IF: immunofluorescence
IGKC: immunoglobulin kappa C
ILC3: group 3 innate lymphoid cell
IL-10: interleukin 10
IRAE: immune-related adverse event
IRF4/8: interferon regulatory factor 4/8
LARS2 B: leucine-tRNA-synthetase-2-expressing B cell
L-kyn: L-kynurenine
LMA: lympho-myeloid aggregate
LN: lymph Node
LT: lymphotoxin
LTB4: leukotrienes B4
LTi: lymphoid tissue inducing cell
LTo: lymphoid tissue organizer cell
LUAD: lung adenocarcinoma
LXA4: lipoxin A4
LXR-Tspan6: liver X receptor-tetraspanin 6
MASH: metabolic dysfunction-associated steatohepatitis
MDSC: myeloid-derived suppressive cell
mIHC: multiplex immunohistochemistry
MZ BC: marginal zone B cell
NK cell: natural killer cell
NPC: nasopharyngeal carcinoma
NSCLC: non-small cell lung cancer
OS: overall survival
PAX5: Paired Box 5
PBMC: peripheral blood mononuclear cell
PDAC: pancreatic ductal adenocarcinoma
PD-L1: programmed death ligand 1
PFS: progression-free survival
PlGF: placental growth factor
PNAd: peripheral node addressin
PPARα: peroxisome proliferator-activated receptor α
Pre-B cell: precursor B cell
Pro-B cell: progenitor B cell
RCC: renal cell carcinoma
RFS: recurrence-free survival
ROS: reactive oxygen species
RNA-seq: RNA sequencing
SHM: somatic hypermutation
SLO: secondary lymphoid organ
ST: spatial transcriptomics
TAA: tumor-associated antigen
Tbet: T-box expressed in T cell
tBreg: tumor-induced Breg
TDLN: tumor-draining lymph node
TEB: tumor-educated B cell
TET2: ten-eleven translocation-2
TIM: T-cell immunoglobulin and mucin-domain
Tfh: T follicular helper cells
Tfr: follicular regulatory T cell
TIB: tumor-infiltrating B cell
TIL: tumor-infiltrating T lymphocyte
TLS: tertiary lymphoid structures
TLR: toll-like receptor
TMB: tumor mutation burden
TME: tumor microenvironment
TNBC: triple-negative breast cancer
TNF: tumor necrosis factor
Tph: T peripheral helper cell
Treg: regulatory T cell
TRAIL: TNF-related apoptosis-inducing ligand
Trm: tissue-resident memory T cell
TSA: tumor-specific antigen
VEGFC: vascular endothelial growth factor C
VTP: vascular targeting peptide
12-CS: 12-chemokine signature
5-hmc: 5-hydroxymethylcytosine
5-LO: 5-lipoxygenase
